# Prospective Trial of Functional Thyrotropin Receptor Antibodies in Graves Disease

**DOI:** 10.1210/clinem/dgz292

**Published:** 2019-12-22

**Authors:** George J Kahaly, Tanja Diana, Michael Kanitz, Lara Frommer, Paul D Olivo

**Affiliations:** 1 Department of Medicine I, Johannes Gutenberg University (JGU) Medical Center, Mainz, Germany; 2 Department of Molecular Microbiology, Washington University Medical School, St. Louis, Missouri

**Keywords:** TSH-R stimulating antibodies, TSH-R blocking antibodies, prospective trial, dilution analysis, Graves hyperthyroidism

## Abstract

**Context:**

Scarce data exist regarding the relevance of stimulatory (TSAb) and blocking (TBAb) thyrotropin receptor antibodies in the management of Graves disease (GD).

**Objective:**

To evaluate the clinical utility and predictive value of TSAb/TBAb.

**Design:**

Prospective 2-year trial.

**Setting:**

Academic tertiary referral center.

**Patients:**

One hundred consecutive, untreated, hyperthyroid GD patients.

**Methods:**

TSAb was reported as percentage of specimen-to-reference ratio (SRR) (cutoff SRR < 140%). Blocking activity was defined as percent inhibition of luciferase expression relative to induction with bovine thyrotropin (TSH, thyroid stimulating hormone) alone (cutoff > 40% inhibition).

**Main Outcome Measures:**

Response versus nonresponse to a 24-week methimazole (MMI) treatment defined as biochemical euthyroidism versus persistent hyperthyroidism at week 24 and/or relapse at weeks 36, 48, and 96.

**Results:**

Forty-four patients responded to MMI, of whom 43% had Graves orbitopathy (GO), while 56 were nonresponders (66% with GO; *P* < 0.01). At baseline, undiluted serum TSAb but not thyroid binding inhibitory immunoglobulins (TBII) differentiated between thyroidal GD-only versus GD + GO (*P* < 0.001). Furthermore, at baseline, responders demonstrated marked differences in diluted TSAb titers compared with nonresponders (*P* < 0.001). During treatment, serum TSAb levels decreased markedly in responders (*P* < 0.001) but increased in nonresponders (*P* < 0.01). In contrast, TBII strongly decreased in nonresponders (*P* = 0.002). All nonresponders and/or those who relapsed during 72-week follow-up period were TSAb-positive at week 24. A shift from TSAb to TBAb was noted in 8 patients during treatment and/or follow-up and led to remission.

**Conclusions:**

Serum TSAb levels mirror severity of GD. Their increase during MMI treatment is a marker for ongoing disease activity. TSAb dilution analysis had additional predictive value.

## Introduction

Graves disease (GD) is characterized by the infiltration of immune effector cells and thyroid-antigen–specific T cells into the thyroid and tissues expressing the thyrotropin receptor (TSH-R), with the production of autoantibodies to well-defined thyroidal antigens (1, 2). The TSH-R expressed on the plasma membrane of thyroid epithelial cells is central to the regulation of thyroid growth and function (3). The TSH-R is the major autoantigen in the autoimmune (4, 5) hyperthyroidism of GD, in which T cells and autoantibodies are directed at the TSH-R antigen. Stimulatory autoantibodies in GD activate the TSH-R, leading to thyroid hyperplasia and unregulated thyroid hormone production and secretion (6).

Currently, 2 different methods of assessing antibodies directed against the TSH-R are being used (7–9). Immunoassays that measure the binding of antibodies to the TSH-R and assays that measure thyroid binding inhibitory immunoglobulin (TBII) quantify the titer of patient’s immunoglobulins that inhibit the binding of thyrotropin (TSH, thyroid-stimulating hormone) to purified or recombinant TSH-R. Such immunoassays thus measure all types of anti-TSH-R antibodies. The second method includes bioassays that can distinguish between stimulatory (TSAb), neutral, and blocking (TBAb) autoantibodies through their effect on cyclic adenosine monophosphate (cAMP) production in a cell line stably transfected with the receptor (10–12). For one such bioassay, a cell line (Mc4-CHO-Luc) was bio-engineered to constitutively express a chimeric TSH-R and a cAMP-inducible luciferase reporter gene that enables quantification of TSAb as a function of the level of luciferase activity (13, 14). The improved sensitivity and accuracy of the TSAb-Mc4 bioassay compared with the previous wild-type TSH-R bioassays is largely attributed to a fully functional leucine repeat domain (15). Using different assay conditions, this Mc4-CHO-Luc cell line measures the activity of TSAb and/or TBAb (16–18).

Unfortunately, a large percentage of patients with GD either do not go into remission or relapse after stopping medical treatment with antithyroid drugs (ATD) (19–22). Therefore, any test that would predict which patients would remain in remission and which patients would relapse before stopping the ATD would be useful (23, 24). Sparse data regarding the clinical relevance of TSAb (25–27) and/or TBAb during GD treatment are available, and the feasibility of the use of bioassays for TSAb and TBAb has not yet been proven in a clinical setting. Therefore, there is a need for a specific biomarker for the management of GD, and evidence suggests that TSAb is a better predictor of patients’ response to ATD treatment than predicate TBII assays. In this study, we aimed to evaluate the clinical utility and predictive value of TSAb and TBAb in the management of GD. We prospectively tested the ability of 2 bioassays for the measurement of functional antibodies to serve as sensitive indices of remission or relapse of GD after treatment with ATD.

## Methods

### Patients and study design

The prospective, single-center trial was approved by the local Ethics Committee (Medical Chamber of the Federal State of Rhineland Palatinate, Mainz, Germany) before initiating the investigation, and all patients provided written informed consent. One hundred well-defined, consecutive, unselected, untreated hyperthyroid patients with GD followed at the tertiary referral center for autoimmune thyroid diseases, JGU Medical Center, were enrolled in a prospective 2-year trial. Patients were asked to participate in the study when hyperthyroidism had been verified and subtyped as GD. Subtyping was based on measurement of TBII positivity in serum and no other cause for the hyperthyroidism was identified. Diagnosis of GD was also suspected on the basis of a typical clinical presentation, the presence of a diffusely enlarged thyroid gland, and evidence of extra thyroidal involvement, if present, and confirmed by the ultrasound image including markedly enhanced perfusion of the thyroid gland on Doppler examination. All patients with GD were screened for signs and symptoms of orbital involvement. Graves orbitopathy (GO) was diagnosed according to the recommendations of the European Group on Graves’ Orbitopathy (28, 29).

Exclusion criteria were age < 18 years, pregnancy within 1 year, severe GO with a need of medical immunosuppressive therapy, intake of drugs affecting the immune system, imminent or manifest thyrotoxic crisis, other severe disease making it unlikely for the patient to be able to follow protocol, thyroid nodules with a need of surgery, intolerance to both methimazole (MMI) and propylthiouracil (PTU), previous surgical or radioactive iodine therapy for GD, patient preference of other type of therapy, patient considered unsuited for this type of therapy by responsible physician, or TBII not measurable at time of diagnosis.

MMI was administered for 24 weeks according to baseline serum concentrations of free triiodothyronine (T3) and free thyroxine (T4). Starting dose ranged from 5 to 30 mg per day. Through a titration regimen, this dose was respectively tapered or increased at each subsequent study visit as the patient became euthyroid or remained hyperthyroid. Goals of therapy were to maintain normal free T4 and free T3 serum levels as well as serum TSH levels in the lower normal range. MMI therapy was stopped at week 24. The main outcome measure was clinical response versus nonresponse to a 24-week MMI treatment, defined as biochemical euthyroidism versus persistent hyperthyroidism at week 24 and further remission within the 72-week follow-up versus persistent hyperthyroidism at week 24 and/or relapse at weeks 36, 48, and 96. Safety was assessed based on treatment‐emergent adverse events observed by caregivers or reported by patients spontaneously or after questioning; these events were rated as related or unrelated to MMI administration.

### Bioassay for stimulating TSH-R-Ab (TSAb)

Serum TSAb concentrations were measured in a blinded manner with a Food and Drug Administration (FDA)-cleared cell-based assay (Thyretain, Quidel, San Diego, CA) according to the manufacturer’s instructions (16, 30–32). Briefly, Chinese Hamster Ovary (CHO)-MC4 cells were seeded and grown to confluent cell monolayers in 96-well plates for 15 to 18 hours. Patient and control samples (required volume 30 µl), as well as positive, reference, and normal controls were diluted 1:11 in reaction buffer (RB, Quidel), the diluted samples were added to the cell monolayers, and each plate was incubated for 3 hours at 37°C with 5% carbon dioxide. Subsequently, the CHO-MC4 cells were lysed and the relative light unit values were quantified in a luminometer (Infinite M200; Tecan, Crailsheim, Germany). The assay cutoff is at a percentage specimen-to-reference-ratio (SRR) of 140%. All sera were measured in duplicates. Data are expressed as mean values.

### Bioassay for blocking TSH-R-Ab (TBAb)

Serum TBAb levels were measured according to the manufacturer’s instructions of the CE-marked cell-based assay (Quidel) (17, 18, 33, 34). The cutoff is 40% Inhibition (I). All sera (required volume 30 µl) were measured in duplicates; data were expressed as mean values.

### Serial dilution analysis of TSH-R-Ab

Serial 1:3 dilutions were performed on each patient serum sample (200 µl) into TSH-R-Ab-negative control serum (400 µl) up to a final dilution of 1:6561 as previously reported (35–37). The titer was defined as the first dilution step at which the assay results fell below the cutoff.

### Thyroid-related hormones, TBII, thyroid peroxidase and thyroglobulin assays

Serum concentrations of thyroid binding inhibitory immunoglobulins (TBII), anti-thyroid peroxidase (TPO) and anti-thyroglobulin (Tg) antibodies, serum TSH, free T3, total T3, free T4, and thyroglobulin were measured with the Cobas e411 analyzer (Elecsys, Roche Diagnostics, Mannheim, Germany) according to the manufacturer’s instructions. All samples were measured within one assay run.

### Statistical analysis

Statistical analysis was done with GraphPad Prism Software, Inc. (version 5.04), (GraphPad, San Diego, CA, USA). All *P* values were two-sided and considered significant when *P* ≤ 0.05. For data showing a Gaussian distribution, the unpaired *t*-test was performed. For not normally distributed data, the Mann-Whitney U test was used. For correlation analyses, the Pearson correlation coefficient was calculated. Positivity rates for each dilution were compared between the different TSH-R-Ab assays by the McNemar test. Univariate and multivariate logistic regression were used to examine the effect of thyroid-related hormones and antibodies, thyroid volume, and the presence of orbitopathy and smoking on response to therapy or recurrence.

## Results

### Clinical data

Baseline demographic, clinical, and serological data of the consecutive, untreated, Caucasian thyrotoxic patients are shown in Table 1. As anticipated, a female-to-male ratio of 4:1 was noted and most patients were in the fourth and fifth decade of life. Patients with new and/or recent onset (< 4 weeks) of Graves hyperthyroidism, patients with GD + GO and nonsmokers were in the majority. Thyroid ultrasound was performed in all patients and showed a typically hypoechoic gland with a strongly enhanced Doppler color flow (“thyroid storm”). With a few exceptions, patients were on beta-blockers (bisoprolol 5 mg per day). Six patients had an isolated free T3/total T3 hyperthyroidism and a free T4 within the normal range.

**Table 1.** Demographic, Clinical and Serological Data

Patients with untreated Graves’ hyperthyroidism	N = 100
Ethnicity/Race	Caucasian/White
Sex, female / male	82 / 18
Smoking, yes / no	41 / 59
Age (median), years (range)	43 (18–78)
Age class, years	
18–39	41
40–59	47
≥ 60	12
Body height, cm	168 (159–184)
Body weight, kg	64.5 (52.5–79)
Body mass index, kg/m^2^	22.9 (20.8–26.0)
Graves thyroidal disease only	N = 44
Graves thyroidal + orbital disease	N = 56
Duration of Graves hyperthyroidism, months	8 (0–37)
Duration of Graves orbitopathy, months	7 (0–31)
New onset / relapse	57 / 43
Isolated free T3 / total T3 hyperthyroidism	6
Beta-blockade medication	91
GREAT score, points	3 (0–6)
Thyroid volume (median), mL (range)	21 (13–64)
TSH (normal range 0.4–4.9 mU/L)	0.01 (0.01-0.01)
Free T4 (0.7–1.5 ng/dL)	2.9 (1.1–8.3)
Free T3 (1.7–3.7 pg/mL)	7.7 (3.8–30)
Total T3 (0.6–1.6 ng/mL)	2.8 (1.7–8.0)
Thyroglobulin (<55 ng/mL)	233 (42–2953)
TPO-Ab (<6 IU/mL)	158 (3.0-1000)
Tg-Ab (<4.1 IU/mL)	5.5 (1.0-1000)
TBII (<1.75 IU/L)	9.8 (1.8–40)
TSAb (<140% SRR)	481 (165–868)
TSAb dilution titer	4 (1–8)
TBII dilution titer	1 (1–3)

Median and range values are shown. GREAT score according to reference 38.

Abbreviations: T3, triiodothyronine; T4, thyroxine; TBII, thyroid binding inhibitory immunoglobulins; Tg-Ab, thyroglobulin antibodies; TPO-Ab, thyro-peroxidase antibodies; TSAb, stimulatory thyrotropin receptor antibodies; TSH, thyrotropin (thyroid stimulating hormone).

#### Efficacy.

Antithyroid treatment with MMI gave a response rate of 44/100 with biochemical euthyroidism at week 24 and sustained remission during a 72-week follow-up period (Table 2). No significant differences were observed between patients with recent onset of GD and those with relapse of their hyperthyroidism. The MMI dosage was titrated at each visit according to the serum levels of free T3 and free T4. At week 24, the median dosage of MMI was 2.5 mg per day and 10 mg/day in the responders and nonresponders, respectively (*P* < 0.001). Responders were followed for 18 months, while nonresponders were again on MMI therapy (median dose 10 mg/day, range 5–20 mg). Significantly more patients with GO (*P* < 0.004), smokers (*P* < 0.01), larger thyroid gland (*P* = 0.002), higher serum free and total T3 concentrations (*P* < 0.001), MMI daily dose (*P* < 0.001), and GREAT scores (38) were registered in nonresponders (Table 3). In univariate analyses, factors associated with relapse of overt hyperthyroidism were high TSAb and TBII levels, thyroid volume, free thyroid hormones, total T3, and smoking. In multivariable analysis, presence of GO (odds ratio [OR] 5.196; *P* < 0.021), 2-fold increased free T3 (OR 1.622; *P* < 0.002), TSAb (OR 1.268; *P* < 0.012), and TBII (OR 1.193; *P* < 0.015) were significant predictors.

**Table 2.** Thyroid-Related Hormones in 100 Patients with Graves Hyperthyroidism Before and After Completion of Methimazole Treatment (week 24) and During a 72-Week (18-month) Follow-Up; Thyro-Peroxidase and Thyroglobulin Antibodies are Shown at Baseline and Week 24

	Week 0	24	36	48	Week 96
Responder (n = 44)					
Free T4 (0.7–1.5 ng/dL)	1.7 (1.1–3.2)	1.1 (0.7–1.4)	1.0 (0.7–1.4)	1.2 (0.9–1.4	1.1 (0.8–1.3)
Free T3 (1.7–3.7 pg/mL)	5.3 (3.8–17)	3.0 (2–3.6)	3.0 (1.8–3.5)	2.7 (1.9–3.4)	2.4 (1.8–3.1)
Total T3 (0.6–1.6 ng/mL)	2.1 (1.7–5.6)	1.3 (1–1.6)	1.1 (0.8–1.5)	1.0 (0.9–1.4)	0.8 (0.6–1.5)
TSH (0.4–4.9 mU/L)	0.01 (0.01–0.01)	0.41 (0.1–0.72)	0.76 (0.31–1.22)	1.34 (0.67–2.1)	1.5 (0.9–2.8)
TPO-Ab (<6 IU/mL)	129 (3.0–1000)	58 (3.0–840)			
Tg-Ab (<4.1 IU/mL)	17.3 (1.0–1000)	10.2 (1.0–1000)			
Nonresponder (n = 56)					
Free T4 (0.7–1.5 ng/dL)	4.2 (2.5–8.3)	3.3 (1.7–6.2)	2.9 (1.5–4.7)	1.4 (0.8–2.7)	1.2 (0.9–1.5)
Free T3 (1.7–3.7 pg/mL)	10.4 (4.2–30)	5.75 (3.8–15.3)	4.9 (3.7–20.5)	3.7 (2.5–8.1)	2.8 (2.1–3.8)
Total T3 (0.6–1.6 ng/mL)	4.0 (2.8–8.0)	3.9 (2.1–7.6)	3.01 (1.7–7.7)	1.6 (1.2–3.3)	1.0 (0.7–1.7)
TSH (0.4–4.9 mU/L)	0.01 (0.01–0.01)	0.01 (0.01–0.01)	0.05 (0.01–0.13)	0.19 (0.08–0.27)	0.24 (0.16–0.38)
TPO-Ab (<6 IU/mL)	183 (3.0–1000)	297 (3.0–1000)			
Tg-Ab (<4.1 IU/mL)	4.3 (1.0–1000)		4.05 (1.0–440)		

Results are shown as median values and range. Responders were off antithyroid treatment after week 24 while nonresponders were further on methimazole (titration regimen) until week 96.

Abbreviations: T3, triiodothyronine; T4, thyroxine; Tg-Ab, thyroglobulin antibodies; TPO-Ab, thyro-peroxidase antibodies; TSH, thyrotropin (thyroid stimulating hormone).

**Table 3.** Clinical and Serological Values at Week 0 in Responders Versus Nonresponders

	Responders	Nonresponders	*P* value
N	44	56	
Thyroidal disease only (n = 44)	25 (57%)	19 (43%)	
Thyroidal + orbital disease (n = 56)	19 (34%)	37 (66%)	<0.004
New onset and untreated	29 (66%)	28 (50%)	
Relapse and previously treated (ATD)	15 (34%)	28 (50%)	
Duration of GD (mo)	6 (0–18)	10 (0–37)	
Age (median), years (range)	42 (20–78)	43.5 (18–74)	
Sex (female / male)	35 (80%) / 9 (20%)	46 (82%) / 10 (18%)	
Smoking (yes / no)	13 (29.5%) / 31 (70.5%)	28 (50%) / 28 (50%)	<0.01
<5 cigarettes/day	(9.6%)	(3.6%)	
6–19 cigarettes/day	(19%)	(37.5%)	
>20 cigarettes /day	2.4%)	(10.9%)	
Thyroid volume (mL)	16 (13–36)	23.5 (15–64)	=0.002
Methimazole dose (mg/d)	7.5 (5–15)	20 (10–30)	<0.001
GREAT score (points)	1(0–2)	4 (3–6)	<0.001
GREAT class	I	III	
TSH (0.4–4.9 mU/L)	0.01 (0.01–0.01)	0.01 (0.01–0.01)	
Free T4 (0.7–1.5 ng/dL)	1.7 (1.1–3.2)	4.2 (2.5–8.3)	<0.001
Free T3 (1.7–3.7 pg/mL)	5.3 (3.8–17)	10.4 (4.2–30)	<0.001
Total T3 (0.6–1.6 ng/mL)	2.1 (1.7–5.6)	4.0 (2.8–8.0)	<0.001
Thyroglobulin (<55 ng/mL)	88 (42–717)	414 (75–2953)	
TPO-Ab (<6 IU/mL)	129 (3.0–1000)	183 (3.0–1000)	
Tg-Ab (<4.1 IU/mL)	17.3 (1.0–1000)	4.3 (1.0–1000)	
TBII (<1.75 IU/L)	6.2 (1.8–28.3)	15.3 (3.1–40)	<0.001
TSAb (<SRR% 140%)	311 (165–445)	573 (389–868)	<0.001
TSAb dilution titer	2 (1–3)	5 (4–8)	<0.001
TBII dilution titer	1 (1–3)	2 (1–3)	

Median and range values or absolute numbers and percentages are shown. Significant *P* values are shown only. GREAT score according to reference 38.

Abbreviations: ATD, antithyroid drugs; GD, Graves disease; T3, triiodothyronine; T4, thyroxine; TBII, thyroid binding inhibitory immunoglobulins; Tg-Ab, thyroglobulin antibodies; TPO-Ab, thyro-peroxidase antibodies; TSAb, stimulatory thyrotropin receptor antibodies; TSH, thyrotropin (thyroid stimulating hormone).

#### Safety.

Seventeen patients experienced adverse effects deemed to be drug‐related, all within the first 3 months. Fourteen patients developed mild skin reactions, 2 other patients had slightly elevated liver enzymes and 1 had mild leukopenia. A switch from MMI to PTU was not necessary. No serious complications, such as agranulocytosis, or unexpected drug‐related adverse events were noted. MMI dose adjustment prevented long-term suppression of serum TSH and no side effects were observed from month 3 through the end of MMI treatment.

### Serology

**Figure 1.**
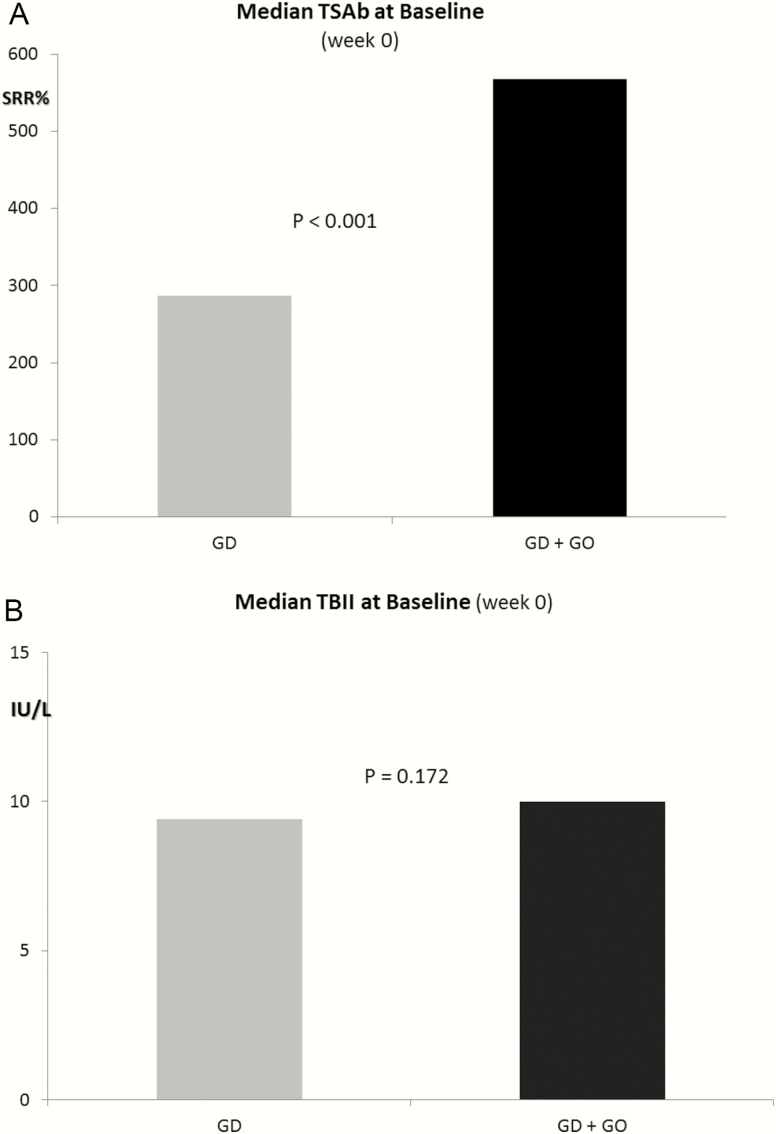
(A) Median serum TSAb concentration (SRR %) at baseline (week 0) prior to methimazole (MMI) treatment in patients with Graves thyroidal disease (GD) only (SRR% range, 165%–391%) and in patients with GD and orbital involvement (GO) (SRR% range, 320%–868%). (B) Median serum TBII concentrations (IU/L) at baseline (week 0) prior to MMI treatment in patients with GD only (range, 1.8–40 IU/L) and in patients with GD + GO) (range, 1.8–40 IU/L).

At baseline, markedly higher serum concentrations of TSAb were noted in patients with GD + GO versus Graves thyroidal disease only (*P* < 0.001) (Fig. 1A). In contrast, no differences were noted for TBII (*P* > 0.05) (Fig.1B). In addition, at baseline, marked differences in diluted serum TSAb titers were registered between responders versus nonresponders (*P* < 0.001) (Table 2). All patients with a TSAb dilution titer > 3 did not respond to MMI treatment. In contrast, TBII dilution titers did not differentiate between responders and nonresponders to MMI therapy and serum samples became TBII-negative at low dilutions.

**Figure 2.**
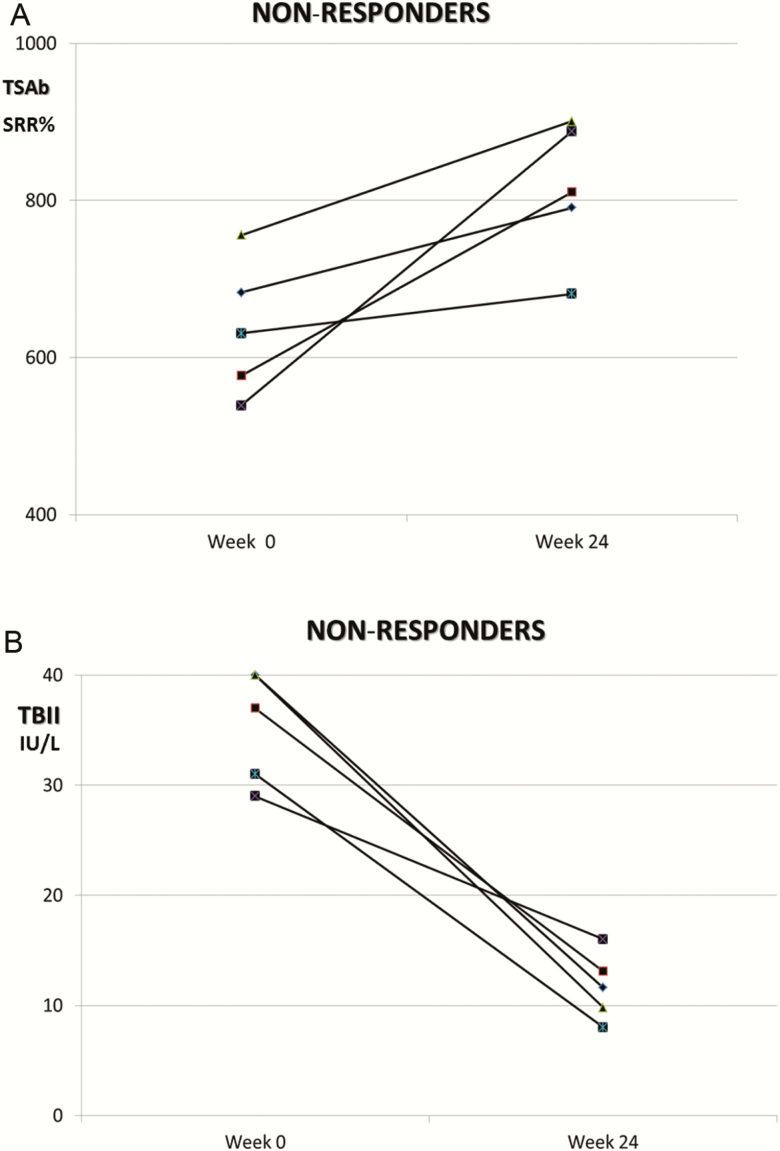
(A) Course of serum TSAb (SRR%) in 5 exemplary patients with Graves hyperthyroidism who did not respond to a 24-week course of methimazole (MMI) treatment. (B) Course of serum TBII (IU/L) in 5 exemplary patients with Graves hyperthyroidism who did not respond to a 24 week of MMI treatment.

As soon as 12 weeks after starting MMI, differences in TSAb levels (*P* < 0.001) were noted between responders versus nonresponders. At week 24 and compared with baseline, serum TSAb levels decreased markedly (−60%; *P* < 0.001) in responders (Table 4), but increased in nonresponders (+17%; *P* < 0.01) (Fig. 2A). In contrast, serum TBII levels decreased in nonresponders (Fig. 2B) at week 24 (−40%; *P* = 0.002). All nonresponders at week 24 and/or those who relapsed during the 72-week follow-up period were TSAb-positive at week 24. Sample dilution analysis during both MMI treatment and follow-up emphasized the difference of clinical sensitivity between functional and binding TSH-R-Ab assays. TSAb positively correlated (all *P* < 0.001) with serum free T3 (*r* = 0.721), free T4 (*r* = 0.699), total T3 (*r* = 0.648), serum TBII levels (*r* = 0.661), and thyroid volume (*r* = 0.697).

**Table 4.** Qualitative and Quantitative Results of the Serum TSH-R-Ab in 100 Patients with Graves Hyperthyroidism Before and After Completion of Methimazole Treatment (week 24) and During a 72-Week (18-month) Follow-Up

	Week 0	24	36	48	96
Responder (n = 44)					
TSAb positive	44 (100%)	37 (84%)	32 (73%)	20 (45%)	0
TSAb (SRR %)	311 (165–445)	151 (65–343)	143 (51–286)	111 (39–213)	
TSAb dilution titer	2 (1–3)	1 (1–2)	1 (0–1)	0 (0–1)	
TBII positive	44 (100%)	26 (59%)	25 (57%)	21 (48%)	0
TBII (IU/L)	6.2 (1.8–28.3)	3.3 (0.5–10.4)	2.9 (0.5–7.1)	1.8 (0.5–2.7)	
TBII dilution titer	1 (1–3)	1 (0–2)	0 (0–1)	0 (0–0)	
TBAb positive	0	4 (9%)	3 (7%)	1 (2%)	0
TBAb (% inhibition)	87 (66–100)	79 (71–92)	68		
TBAb dilution titer	3 (2–5)	2 (1–3)	1		
Nonresponder (n = 56)					
TSAb positive	56 (100%)	56	56	56	56 (100%)
TSAb (SRR %)	573 (389–868)	698 (501–913)	551 (311–676)	393 (199–508)	214 (143–279)
TSAb dilution titer	5 (4–8)	5 (4–7)	4 (3–7)	4 (3–6)	3 (2–4)
TBII positive	56 (100%)	52 (93%)	47 (84%)	39 (70%)	28 (50%)
TBII (IU/L)	15.3 (3.1–40)	9.3 (1.8–16.2)	5.6 (1.75–8.9)	4.3 (0.5–7.2)	2.0 (0.5–2.6)
TBII dilution titer	2 (1–3)	1 (0–2)	1 (0–2)	1 (0–1)	(0–1)
TBAb positive	0	0	0	0	0

Qualitative TSAb, TBAb, and TBII results are stated as absolute values and percentage. Quantitative serum concentrations for TSAb, TBAb, and TBII, as well as TSAb and TBII dilution titers are given as median values and range. Cutoff values for TSAb, TBAb, and TBII are SRR 140%, 40% inhibition, and 1.75 IU/L, respectively.

Abbreviations: SRR, specimen-to-reference ratio; TBAb, blocking thyrotropin receptor antibodies; TBII, thyroid binding inhibitory immunoglobulins; TSAb, stimulatory thyrotropin receptor antibodies.

A shift from TSAb to TBAb (Table 4) and/or dual positivity for TSAb and TBAb during MMI treatment or after completion of ATD therapy was noted in 8 GD patients (4 at week 24, 3 at week 36, and 1 at week 48) and was associated with clinical and biochemical remission. Compared with TSAb, TBAb positivity was observed in the responder group only and was transient occurring within a range of 3 to 9 months. Declining serum TBAb dilution titers were noted during treatment and follow-up.

## Discussion

This report describes a prospective trial sequentially evaluating a large collective of consecutive, untreated hyperthyroid patients with GD prior, during and after completion of ATD treatment and demonstrates the clinical relevance and utility of functional TSH-R-Ab. Indeed, TSAb were a useful biomarker for activity, severity, and/or systemic involvement of GD. Compared with TBII, TSAb levels were a better early predictor of disease progression, remission, or response to therapy. Thus, serum TSAb levels mirrored the severity of GD. Their increase during treatment was a marker for ongoing disease activity. In addition, using serial TSAb dilution analysis, TSAb titers were predictive for response to ATD treatment with 100% nonresponse and/or relapse in the case of a baseline TSAb titer > 3. Furthermore, and in contrast to the baseline TBII levels, baseline serum TSAb concentrations significantly differentiated between GD thyroidal disease only and GD + GO. Finally, TSAb positivity independently predicted GD relapse in the multivariate analysis. Thus, TSAb levels are a prognostic parameter for GD and reliably predict response to medical therapy.

Sequential measurement of the blocking TSH receptor antibody during and after treatment was also useful, since appearance of this antibody was associated with clinical and biochemical remission. Relative to the sustained positivity of TSAb in nonresponders and patients with severe thyrotoxicosis, TBAb was rather transient, being present for 3 to 9 months with declining serum levels. Therefore, a shift from TSAb to TBAb seems to impact the clinical course of the disease. This observation supports the use of a “functional TSH-R-Ab package,” which can provide more relevant information for the monitoring and follow-up of patients with GD.

The prediction of response to therapy in GD after a course of ATD treatment remains a challenge for individual patient management. To this end, many studies have evaluated the use of TBII with conflicting results (24, 39–43). Although TBII determination based on second- and third-generation assays has superior diagnostic quality, no clinically valuable outcome prediction is seen at various diagnostic cutoffs (24, 41, 44). According to the later reports, high TBII levels are relevant predictive values of relapse of GD, either during ATD therapy or after ATD withdrawal. Nevertheless, to date, it is still unclear why TBII is such an excellent tool for the diagnosis of GD but performs rather poorly in the prediction of relapse. In comparison, this prospective trial has demonstrated the predictive role of the functional stimulatory TSH-R-Ab as a clear indicator and biomarker of persistent active Graves disease. Indeed all patients who did not respond at week 24 were TSAb-positive. Hence the magnitude of the SRR% and the qualitative serum TSAb positivity are predictive for a nonresponse. On the other hand, a low positive SRR% at week 24 does not exclude a relapse during follow-up. Furthermore, the TSAb dilution analysis at baseline demonstrated that all patients whose samples were still positive at dilution step 3 to 4 did not go into remission. The cutoff of 4 dilutions was a more reliable indicator for nonresponse and/or relapse when compared with the baseline undiluted SRR%. Of note, dilution analysis at baseline for TBII or TSH-R-Ab (conventional binding assay) was neither informative nor helpful.

To this end, it is generally accepted that TSH-R-Ab are heterogeneous, usually existing as a mixture of antibodies binding to different epitopes of the TSH-R, and are further complicated by the possibility of the recognition of variable epitopes during the course of the disease (45). However, TSAb and TBAb may not be entirely different in terms of epitope recognition and affinities to the TSH-R. Work on monoclonal antibodies found comparable displacement between TSAb and TBAb monoclonal antibodies in patient serum (46, 47). Studies using purified antibodies from patients with GD suggest that TSAb antibodies from hyperthyroid patients and TBAb antibodies from hypothyroid patients share close epitopes on the TSH-R, and have comparable affinity (48).

In line with this, functional bioassays that measure cAMP production (49–51) and/or luciferase expression (15, 16, 31) in response to TSH-R interaction with TSAb could improve the detection of TSH-R-Ab in patients with GD. Also, functional bioassays, which determine the effect of TSH-R activation rather than just the binding of antibodies, may improve the prediction of relapse. Further, determining a change in the balance between stimulating and blocking activities may offer a possible explanation for the fluctuation of thyroid function observed in certain patients (52, 53). Finally, demographic considerations may be important. For example, Asian patients with GD are more likely to develop hypothyroidism after treatment, which is possibly correlated with a higher prevalence of blocking antibodies (54).

In conclusion, this prospective 2-year trial demonstrates the relevance, clinical utility and most importantly, the predictive value of functional thyrotropin receptor antibody measurement in the management of patients with Graves hyperthyroidism.

## Abbreviations

ATD: antithyroid drugs
cAMP: cyclic adenosine monophosphate
GD: Graves disease
GO: Graves ophthalmopathy
MMI: methimazole
PTU: propylthiouracil
SRR: specimen-to-reference ratio
T3: triiodothyronine
T4: thyroxine
TBAb: blocking thyrotropin receptor antibodies
TBII: thyroid binding inhibitory immunoglobulins
TPO: thyroid peroxidase
TSAb: stimulatory thyrotropin receptor antibodies
TSH: thyrotropin (thyroid stimulating hormone)
TSH-R: thyrotropin receptor

## Additional Information

***Disclosure Summary:*** T.D., M.K., and L.F. have nothing to disclose. G.J.K. and P.D.O. consult for Quidel and the JGU Medical Center has received research-associated funding from Quidel.

***Data Availability:*** All data generated or analyzed during this study are included in this published article or in the data repositories listed in References.
